# Multiscale classification of heart failure phenotypes by unsupervised clustering of unstructured electronic medical record data

**DOI:** 10.1038/s41598-020-77286-6

**Published:** 2020-12-07

**Authors:** Tasha Nagamine, Brian Gillette, Alexey Pakhomov, John Kahoun, Hannah Mayer, Rolf Burghaus, Jörg Lippert, Mayur Saxena

**Affiliations:** 1 Droice Research, New York, NY USA; 2 Department of Surgery, NYU Langone Hospital Long Island, Mineola, NY USA; 3 Department of Foundations of Medicine, NYU Long Island School of Medicine, Mineola, NY USA; 4 Clinical Informatics, CityMD, New York, NY USA; 5 Clinical Pharmacometrics, Bayer AG, Wuppertal, Germany

**Keywords:** Heart failure, Machine learning

## Abstract

As a leading cause of death and morbidity, heart failure (HF) is responsible for a large portion of healthcare and disability costs worldwide. Current approaches to define specific HF subpopulations may fail to account for the diversity of etiologies, comorbidities, and factors driving disease progression, and therefore have limited value for clinical decision making and development of novel therapies. Here we present a novel and data-driven approach to understand and characterize the real-world manifestation of HF by clustering disease and symptom-related clinical concepts (complaints) captured from unstructured electronic health record clinical notes. We used natural language processing to construct vectorized representations of patient complaints followed by clustering to group HF patients by similarity of complaint vectors. We then identified complaints that were significantly enriched within each cluster using statistical testing. Breaking the HF population into groups of similar patients revealed a clinically interpretable hierarchy of subgroups characterized by similar HF manifestation. Importantly, our methodology revealed well-known etiologies, risk factors, and comorbid conditions of HF (including ischemic heart disease, aortic valve disease, atrial fibrillation, congenital heart disease, various cardiomyopathies, obesity, hypertension, diabetes, and chronic kidney disease) and yielded additional insights into the details of each HF subgroup’s clinical manifestation of HF. Our approach is entirely hypothesis free and can therefore be readily applied for discovery of novel insights in alternative diseases or patient populations.

## Introduction

Heart failure (HF) is a leading cause of death and morbidity worldwide and is responsible for a large portion of healthcare and disability costs every year^1^. HF is challenging to treat because it can have various causes, is impacted by a wide array of patient genetic and lifestyle factors and comorbidities, and can manifest, progress, and respond to treatment differently among individuals^1–3^. Classification schemes for HF help clinicians determine the disease phenotype, select appropriate treatments, and define study populations for randomized controlled trials (RCT) of HF interventions. Such schemes are typically defined in a top-down manner based on HF etiology^4–6^, functional assessments (e.g., the New York Heart Association Functional Classification), imaging-based lab values (such as ejection fraction) and biomarkers (e.g. NT-proBNP, cardiac troponin)^1,3,7^. However, there is a consensus that these existing classification schemes for HF are coarse and often do not account for the heterogeneity stemming from a wide range of patient factors and comorbidities which may have a large impact on outcomes^3,8^.

Although HF classifications have provided the framing and structure of most research in the field, the development of novel, data-driven classifications of disease that capture heterogeneous clinical presentations have the potential to improve the understanding and care of HF patients, in particular to implement precision medicine and drive better outcomes^9,10^. With the rise of real-world data (RWD) and machine learning, a new line of research has developed that attempts to use these tools to inform these existing schemas^11,12^. The increasing availability of electronic health records (EHR) supplies valuable RWD for analysis of HF subpopulation characteristics and treatment performance, which can provide insights in care settings that may not be accurately represented by the highly controlled care and selective inclusion/exclusion criteria of RCTs. Importantly, EHRs contain is a wealth of rich, real-world information about patient disease captured in the expressive nature of unstructured clinical narratives, in particular regarding the diversity of the conditions in multimorbid patients and patient-reported or non-billable symptoms^13,14^, which are typically not used in existing classification schemes and phenotyping algorithms. This can be of particular interest in heart failure and especially HF with preserved ejection fraction (HFpEF), where there is recognition of a large amount of phenotypic diversity and a lack of interventions shown to improve outcomes^15^.

At the same time, there is a growing body of work that aims to use a data-driven approach to phenotype disease in a variety of conditions, including chronic obstructive pulmonary disease (COPD)^16,17^, asthma^18^, sepsis^19^, gout^20^, Parkinson’s disease^21^, and heart failure^22–24^. In particular, clustering has emerged as a paradigm for inferring phenotypes from data, rather than relying on top-down classification schemes^17,20–22,24,25^. So far, most clustering studies were performed on a limited, fixed set of top-down clinical or domain specific definitions (e.g., specific biomarkers or diagnostic codes) to make the problem of finding subpopulations tractable. This hypothesis driven approach may limit their ability to fully capture the diversity of the disease states of the population and ultimately prevent discovery of novel factors contributing to phenotypic variation. We thus theorized that a data-driven, unsupervised phenotype discovery methodology that groups HF patients according to similarity of disease manifestation (phenotype) in RWD unstructured clinical text can provide insights into HF subpopulations’ disease etiology and defining characteristics that may not be apparent in classically defined HF subgroups.

In this study, we present a hypothesis-free, data-driven clustering approach to understand real-world manifestations of heart failure in a large population of HF patients. Specifically, we construct groups of similar patients via unsupervised clustering of HF patients’ symptoms and complaints mentioned in unstructured EHR data. These clusters and their distinctive pattern of disease manifestation (i.e., clinical complaints) can be understood as HF patient disease phenotypes. Importantly, we find that the resultant HF phenotypes correspond to clinically meaningful etiologies and endpoints of heart failure, which can be interpreted within a hierarchical framework and explored at various levels of granularity. In particular, the ability to reconstruct the pattern of disease subtypes can be beneficial in understanding the diversity of real-world patient populations in complex syndromes like HF. Such an approach can provide a complementary perspective to HF and may ultimately inform and contribute to a more precise HF classification scheme, especially if applied to very large heart failure populations. Finally, because the method is entirely unsupervised and does not require HF-specific domain expertise or definitions, this general methodology can be readily applied to gain insights into real-world manifestations of other complex diseases.

## Materials and methods

In this study, we employed a clustering methodology to partition a large HF population into groups of similar patients. Using statistical testing to find significantly overrepresented patient complaints in the resultant clusters allows these HF patient subgroups to be interpreted as data-driven HF phenotypes. This section presents the methods employed to construct and interpret cluster-based HF phenotypes using a large repository of EHRs.

### Description of dataset

#### Electronic health record dataset

In this study, we used the EHRs from a national medical research center located in a major metropolitan center in western Russia^14^. The center provides the full cycle of medical services, including inpatient and outpatient departments, imaging, rehabilitation services, perinatal care (including pediatric intensive care and surgery), and dentistry. Inpatient services are spread across various institutes and departments, and include, among others, internal medicine, functional diagnostics, intensive care units (ICU), including neonatal ICU (NICU), surgery (including cardiovascular, oncology, neurology, robotic surgery, etc.), clinical pharmacology, and chemotherapy. The longitudinal records used in this study were collected over a 10-year time span (2008–2018). Use of de-identified data for research purposes was approved by the institution.

#### Heart failure cohort definition

The heart failure analysis cohort was defined using the International Classification of Diseases, 10th Revision, Clinical Modification (ICD-10-CM). We included any patient who was diagnosed with an ICD-10 code for heart failure (I50), cardiomyopathy (I42), or hypertensive disease with heart failure (I11.0, I13.0, and I13.2). Patients of any age were included in the cohort. Except for the HF diagnosis no a priori inclusion/exclusion criteria were used.

**Figure 1:**
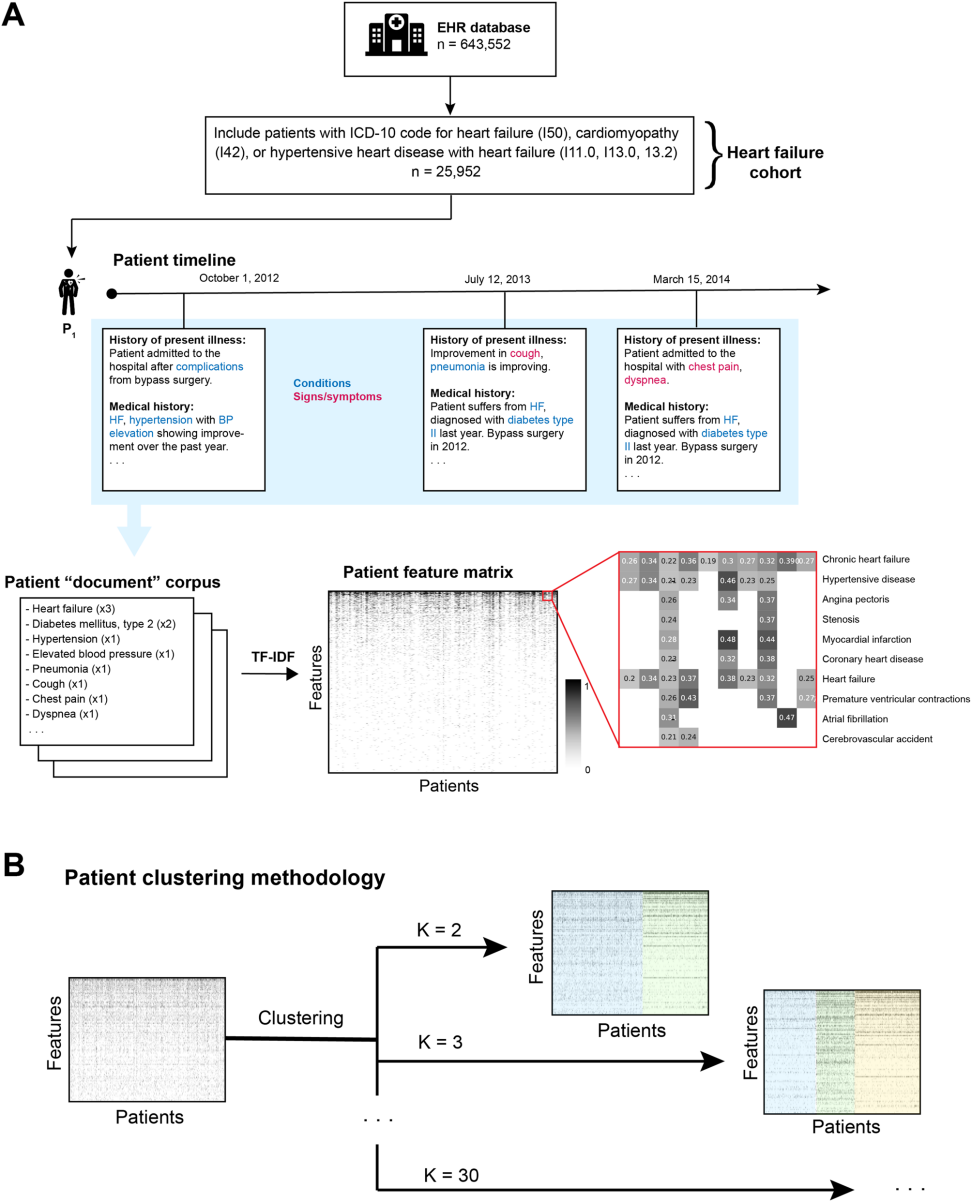
Workflow diagram depicting cohort definition and vectorization of EHRs. (**A**) The final cohort consists of 25,952 individuals with heart failure. An NER system was used to extract condition and symptom mentions in the clinical notes. These were aggregated over the entire timeline of each patient. The resultant “corpus” (medical concept counts for each patient) was then transformed using TF-IDF to obtain a vector space representation of patient EHRs. (**B**) Schematic of patient EHR clustering methodology. The patient-feature matrix derived from patients’ clinical notes in (**A**) was used to fit K-means clustering models for values of K in [2, 3, …, 30]. Examples of clustering results are shown at right for K in [2, 3], with cluster assignment highlighted with colored overlays.

After applying the diagnostic inclusion criteria, the resultant heart failure cohort consisted of 25,952 patients (Fig. 1). The number of patients matching each ICD-10 code in the inclusion criteria is shown in Table 1. A majority of the cohort (79.12%) had an ICD-10 code for heart failure (I50), while 26.24% had a cardiomyopathy code (I42) and 13.59% had a hypertensive heart disease with heart failure code. A majority of the cohort was male (57.4%), and the median age of adults in the dataset was 58 and 63 for males and females, respectively (48, 67 interquartile range for male and 48, 72 female patients), which suggests a relatively young heart failure population and is consistent with expected values of life expectancy and cardiovascular mortality and morbidity in the Russian Federation^26–29^. Table 1 also characterizes the incidence of selected comorbid conditions within the cohort. Patients were labeled with comorbid phenotypes using an ICD-10 code and text-based approach^14^.

**Table 1:** Baseline characteristics of the heart failure cohort.

**Database characteristics**	
Patients in cohort	25,952 (100%)
Unique patients with Hypertensive heart disease with heart failure ICD-10 inclusion criteria (I11.0, I13.0, I13.2)	3,527 (13.59%)
Unique patients with Cardiomyopathy ICD-10 inclusion criteria (I42)	6,811 (26.24%)
Unique patients with Heart failure ICD-10 inclusion criteria (I50)	20,534 (79.12%)
Total number of medical concept mentions	12,490,330
Number of unique medical concepts	1,276
**Patient co-morbidities**	
Congestive heart failure	25,870 (99.68%)
Cardiomyopathy	9,041 (34.84%)
Hypertension	21,933 (84.51%)
Ischemic heart disease	21,358 (82.30%)
Cerebral ischemia	14,633 (56.38%)
Cardiac valve disease	19,960 (76.91%)
Atrial fibrillation and flutter	8,872 (34.19%)
Chronic obstructive pulmonary disease	5,855 (22.56%)
Obesity	9,351 (36.03%)
Hyperlipidemia	14,139 (54.48%)
Type 2 diabetes	5,746 (22.14%)
Chronic kidney disease	3,249 (12.52%)
**Patient characteristics**	
N females	13,220 (42.60%)
Age of males (years), median (25, 75 quartile)	58 (48,67)
Age of females (years), median (25, 75 quartile)	63 (48, 72)
Age of males, 18 + (years), median (25, 75 quartile)	60 (52, 68)
Age of females, 18 + (years), median (25, 75 quartile)	65 (55, 73)
Timeline length (months), median (25, 75 quartile)	4 (1, 28)
BMI, median (25, 75 quartile)	27.34 (23.45, 31.23)
Median number of concepts/patient (25, 75 quartile)	253 (103, 513)
Median number of concepts/patient (unique) (25, 75 quartile)	72 (43, 107)

### Discovering heart failure phenotypes via clustering

#### EHR processing and feature extraction

To cluster patients into HF phenotypes, we first needed to convert the patients in the HF cohort into a vectorized representation suitable as input to a clustering algorithm. We chose to use the clinical notes found in each patient’s EHR as the data source for clustering, since unstructured clinical narratives contain detailed textual descriptions of a patient’s diagnoses, comorbid conditions, unbilled complaints, and other rich descriptors of disease that are often missing from structured data elements.

We extracted complaints from all of the unstructured text in each patient’s EHR for analysis. Medical concepts from the clinical notes of the heart failure patient cohort were identified using the Russian-language clinical named entity recognition (NER) system as described in Ref.^14^ (Fig. 1A). This system extracts mentions of clinical concepts from several clinically relevant ontologies included in the Unified Medical Language System (UMLS)^30^ and maps them to a concept unique identifier (CUI), which allows different strings to be matched to the same concept (e.g., “Type 2 diabetes” and “DM2” will both be assigned the same CUI). In this study, we extracted entities in SNOMED CT^31^ and represented each entity by its normalized CUI.

Within UMLS, each CUI has one or more semantic types. We limited our analysis to entities corresponding to patient *complaints* (e.g., diseases, signs, symptoms, conditions; for a full list of UMLS semantic types, see Supplementary Table S1) and discarded entities corresponding to interventions (e.g., medications, procedures) and anatomy. We also removed from the analysis all entities with negative polarity (e.g., “Patient denies headache”). We aggregated all positively mentioned complaints for each patient over the entirety of the EHR timeline to generate a vector of counts of each complaint for each patient.

The vector of counts of complaints of each patient was then transformed using the term frequency-inverse document frequency (TF-IDF) approach, a standard method for text representation in information retrieval and other NLP algorithms. TF-IDF provides a measure of importance of a word or term to a document within a corpus^32^. The TF for a given complaint counts the number of times that the complaint occurs within the entire EHR of the patient (document), while the IDF penalizes terms that occur in many patients in the cohort (corpus).

#### Inferring HF phenotypes using clustering

We defined heart failure phenotypes by grouping aggregated patient complaint TF-IDF vectors into clusters using K-means clustering^33^ (Fig. 1B). The resultant clusters contain patients grouped by similar patterns of complaints and comorbidities, which can then be interpreted as phenotypes. We applied K-means clustering for $K\epsilon [\mathrm{2,3},\dots ,30]$ and utilized a cluster bootstrapping method to determine the values of $K$ resulting in stable clusters, which can then be interpreted as reproducible, data-driven HF phenotypes.

After finding a set of viable clusters via cluster bootstrapping, we aimed to visualize the hierarchical structure of the clustering result. The resulting phenotype dendrogram allows us to understand the hierarchical relationship between clusters at different values of $K$ and provides a visualization of the phylogenetic tree of complaints and symptoms.

To create a clinical interpretation of each phenotype cluster, we used statistical testing to find complaints that were significantly overrepresented within the cluster as compared to the rest of the heart failure cohort. Doing so allows us to determine the distinguishing medical concepts, or features, associated with each cluster. We employed Bonferroni correction for multiple comparisons. Samples were tested and confirmed to be normally distributed. Finally, we performed an analysis to quantify the co-occurrence rate of important (significantly overrepresented) concepts associated with each cluster. To quantify the co-occurrence of concepts associated with each phenotype, we considered the top 10 most significantly associated (smallest *p* value) concepts in each cluster. Thus, for a given value of $K$, we consider $10K$ concepts; we then calculate a *concept association score* ${a}_{ij}$ using the Jaccard index.

## Results

K-means clustering of the medical complaint vectors of the HF cohort for $K\epsilon [\mathrm{2,3},\dots ,30]$ revealed stable clusters for $K=$ $[\mathrm{2,3},\mathrm{4,6},\mathrm{8,10,13,15,17,27}]$ via cluster bootstrapping (starred values of K in Supplementary Fig. S1), which we consider the hierarchy of data-driven HF phenotypes. In the following sections, we visualize and describe in depth the resultant phenotypes for one value of K (K = 15) as well as interpreting these phenotypes in context of the overall derived data-driven hierarchy of HF.

### Discovering complaints-driven heart failure phenotypes

Table 2 shows the top ten most over-represented complaints and symptoms (smallest *p* values) in each respective HF phenotype cluster for K = 15. We further characterized each cluster by examining descriptive statistics of several clinical characteristics (Table 2). The clinical characteristics we used were number of patients, patient age, sex, body mass index (BMI), in hospital mortality rate, and structured diagnosis codes (ICD-10). For age we used the patient’s age at his or her last encounter. For in hospital mortality rate, we utilized a structured data field in the EHR; actual mortality rates are almost certainly higher. The names chosen for the different clusters reflect the significantly overrepresented complaints and in the respective cluster, as well as the descriptive statistics for each cluster.

**Table 2:** Cluster characteristics for K = 15. Top ten most significant concepts for each phenotype, ranked by *p* value (smallest to largest).

Cluster name	Top ten significant concepts	Descriptive statistics	ICD-10
Cardiac surgery (male)	Cardiac index (90.6%) Peristalsis (95.3%) Cerebrovascular Disorders (72.6%) Color of urine (98.2%) Postpericardiotomy Syndrome (58.6%) Central venous pressure finding (91.4%) Coronary Artery Disease (61.7%) Effusion (76.9%) Cardiac activity (79.3%) Pulmonary artery pressure (78.5%)	N = 2633 20.6% female Age: 63.3 years (57.3, 68.8) BMI: 28.0 (25.5, 31.1) Mortality: 1.21%	I50: Heart failure (98.3%) I20: Angina pectoris (97.0%) I25: Chronic ischemic heart disease (61.1%) I60–I69: Cerebrovascular diseases (45.9%) K20–K31: Diseases of esophagus, stomach and duodenum (23.0%) I70–I79: Diseases of arteries, arterioles and capillaries (21.8%) E08–E13: Diabetes mellitus (13.9%) I21: Acute myocardial infarction (7.40%)
History of myocardial infarction (male)	Hypertensive disease (97.3%) Angina Pectoris (98.3%) Stenosis (87.2%) Myocardial Infarction (78.2%) Coronary heart disease (99.8%) Myocardial Ischemia (96.2%) Systemic arterial pressure (74.3%) Heart failure (98.1%) Atherosclerosis (76.3%) Chronic gastritis (71.3%)	N = 2633 30.1% female Age: 62.5 years (56.1, 68.4) BMI: 28.9 (25.8, 32.2) Mortality: 0.30%	I50: Heart failure (96.8%) I20: Angina pectoris (91.8%) I25: Chronic ischemic heart disease (61.9%) K20-K31: Diseases of esophagus, stomach and duodenum (42.5%) I70-I79: Diseases of arteries, arterioles and capillaries (20.6%) E08–E13: Diabetes mellitus (16.6%)
Acute myocardial infarction (male)	Acute myocardial infarction (91.6%) Myocardial Infarction (99.1%) Acute Coronary Syndrome (82.1%) Coronary heart disease (99.0%) Myocardial Ischemia (96.9%) Infarction (71.8%) Sinus rhythm (95.4%) Akinesia (74.7%) Stenosis (87.1%) Systemic arterial pressure (83.5%)	N = 1227 26.6% female Age: 61.5 years (53.4, 69.3) BMI: 27.6 (24.8, 31.1) Mortality: 3.01%	I50: Heart failure (98.2%) I21: Acute myocardial infarction (91.0%) I25: Chronic ischemic heart disease (45.9%) I20: Angina pectoris (37.3%) K20–K31: Diseases of esophagus, stomach and duodenum (29.9%) I22: Subsequent ST elevation (STEMI) and non-ST elevation (NSTEMI) myocardial infarction (16.2%)
Unstable angina (male)	Unstable Angina (99.2%) Angina Pectoris (96.1%) Myocardial Ischemia (95.3%) Coronary heart disease (98.6%) Acute Coronary Syndrome (71.0%) Progressive Angina (49.8%) Sinus rhythm (91.3%) Hepatitis B (54.8%) Stenosis (80.1%) Pain (86.6%)	N = 1382 36.4% female Age: 64.5 years (57.2, 72.7) BMI: 28.4 (25.2, 32.3) Mortality: 0.50%	I20: Angina pectoris (98.6%) I50: Heart failure (96.4%) I25: Chronic ischemic heart disease (43.4%) K20–K31: Diseases of esophagus, stomach and duodenum (28.5%) I10–I16: Hypertensive diseases (16.4%) E08–E13: Diabetes mellitus (15.6%) I21: Acute myocardial infarction (14.5%)
Congenital heart defects	Congenital Heart Defects (89.0%) Congenital heart disease (99.6%) Congenital Abnormality (96.4%) Birth (89.6%) Pregnancy (85.4%) Air Embolism (65.3%) Atrial Septal Defects (66.2%) Respiration Disorders (56.5%) Childbirth (61.1%) Systolic Murmurs (66.2%)	N = 1599 54.2% female Age: 2.60 years (0.86, 8.56) BMI: 15.7 (14.4, 18.0) Mortality: 0.56%	Q20–Q28: Congenital malformations of the circulatory system (98.4%) I50: Heart failure (97.1%) G96: Other disorders of central nervous system (16.6%) Q90: Down syndrome (7.00%) K20–K31: Diseases of esophagus, stomach and duodenum (6.50%) E40–E46: Malnutrition (6.37%)
NICU	Diuresis (99.0%) Congenital heart disease (97.7%) Birth (98.6%) Congenital Abnormality (94.2%) Newborn (87.0%) Systolic Murmurs (92.0%) Childbirth (87.9%) Wheezing (92.2%) Surgical wound (76.7%) Pregnancy (91.2)	N = 803 41.7% female Age: 0.20 years (0.09, 0.98) BMI: 13.4 (12.1, 15.2) Mortality: 16.6%	I50: Heart failure (98.3%) Q20–Q28: Congenital malformations of the circulatory system (98.2%) G96: Other disorders of central nervous system (32.2%) P50–P61: Hemorrhagic and hematological disorders of newborn (30.5%) P91: Other disturbances of cerebral status of newborn (29.8%) G93: Other disorders of brain (29.2%)
Atrial fibrillation	Atrial Fibrillation (95.7%) Atrial fibrillation and flutter (68.7%) Paroxysmal atrial fibrillation (60.4%) Premature ventricular contractions (87.9%) Atrial Flutter (53.1%) Cardiac Arrhythmia (85.2%) Premature Cardiac Complex (71.8%) Dyspnea (93.1%) Supraventricular arrhythmia (54.6%) Persistent atrial fibrillation (37.9%)	N = 1765 43.2% female Age: 67.1 years (59.3, 75.5) BMI: 29.3 (25.7, 33.4) Mortality: 0.39%	I48: Atrial fibrillation and flutter (68.9%) I50: Heart failure (67.9%) I25: Chronic ischemic heart disease (39.5%) I20: Angina pectoris (36.1%) I10–I16: Hypertensive diseases (31.0%) I42: Cardiomyopathy (23.1%)
Decompensated CHF (male)	Decompensation (63.3%) Pulmonary Hypertension (78.8%) Swelling (80.4%) Pulmonary Embolism (53.2%) Cardiac asthma (49.3%) Hydrothorax (52.4%) Diuresis (87.2%) Ascites (45.3%) Thromboembolism (43.9%) Pulmonary Thromboembolisms (44.2%)	N = 2158 31.1% female Age: 62.8 years (53.2, 71.0) BMI: 27.1 (23.5, 31.1) Mortality: 14.5%	I50: Heart failure (88.7%) I25: Chronic ischemic heart disease (41.8%) I20: Angina pectoris (33.0%) I42: Cardiomyopathy (26.2%) I47: Paroxysmal tachycardia (22.2%) I48: Atrial fibrillation and flutter (17.8%)
Dilated cardiomyopathy (male)	Dilated Cardiomyopathy (97.2%) Chronic heart failure (97.1%) Cardiomyopathies (81.4%) Dyspnea (90.6%) Hypokinesia (73.9%) Mitral Valve Insufficiency (82.8%) Tricuspid Valve Insufficiency (76.0%) Cardiomegaly (36.4%) Myocarditis (30.3%) Ventricular Tachycardia (37.7%)	N = 1597 21.6% female Age: 54.5 years (45.9, 62.3) BMI: 28.0 (25.1, 31.5) Mortality: 0.62%	I42: Cardiomyopathy (93.9%) I50: Heart failure (24.8%) I47: Paroxysmal tachycardia (21.7%) I25: Chronic ischemic heart disease (16.8%) I48: Atrial fibrillation and flutter (11.1%) I20: Angina pectoris (10.7%)
Aortic valve disease	Calcinosis (80.3%) Heart Neoplasm (76.1%) Aortic Valve Stenosis (74.6%) Aortic Valve Insufficiency (88.9%) Heart valve disease (76.7%) Color of urine (85.5%) Cardiac index (74.1%) Central venous pressure finding (76.1%) Cardiac activity (71.3%) Blood flow (96.4%)	N = 1857 49.5% female Age: 66.4 years (57.7, 74.3) BMI: 27.1 (24.2, 30.5) Mortality: 3.50%	I50: Heart failure (93.7%) I35: Nonrheumatic aortic valve disorders (64.0%) I60–I69: Cerebrovascular diseases (41.0%) I20: Angina pectoris (27.4%) I05–I09: Chronic rheumatic heart diseases (26.3%) K20–K31: Diseases of esophagus, stomach and duodenum (23.1%)
Hypertensive heart disease (female)	Heart Diseases (95.8%) Hypertensive disease (97.2%) Heart failure (98.8%) Hyperlipidemia (43.1%) Increase in blood pressure (35.9%) Obesity (46.4%) Menopause present (33.2%) Lipid Metabolism Disorders (25.8%) Gynecological history (24.3%) Vertebrobasilar Insufficiency (22.1%)	N = 1727 72.3% female Age: 63.5 years (55.2, 71.7) BMI: 30.1 (27.4, 35.1) Mortality: 0.05%	I10–I16: Hypertensive diseases (97.5%) I20: Angina pectoris (8.80%) I25: Chronic ischemic heart disease (5.90%)
Cerebrovascular disease	Encephalopathies (67.2%) Dysarthria (52.5%) Gagging (50.8%) Corneal Reflexes (40.0%) Nystagmus (34.7%) On examination—pupil reaction to light (34.7%) Dysphonia (32.6%) Deglutition Disorders (31.5%) Cataract (32.5%) Headache (45.6%)	N = 2348 58.2% female Age: 70.1 years (62.4, 77.6) BMI: 29.0 (25.5, 32.9) Mortality: 0.89%	I50: Heart failure (59.1%) I10–I16: Hypertensive diseases (55.5%) I20: Angina pectoris (51.5%) I60–I69: Cerebrovascular diseases (45.7%) I25: Chronic ischemic heart disease (44.2%) E08–E13: Diabetes mellitus (27.1%)
Hypertrophic cardiomyopathy	Hypertrophic Cardiomyopathy (100%) Left Ventricular Hypertrophy (73.2%) Hypertrophy (45.5%) Hypertrophic cardiomyopathy without obstruction (27.1%) Mitral Valve Insufficiency (77.1%) Diastolic dysfunction (55.3%) Pulmonary Valve Insufficiency (48.5%) Asymmetric hypertrophy (13.5%) Heart murmur (34.0%) Tricuspid Valve Insufficiency (67.9%)	N = 1159 53.9% female Age = 56.9 years (46.7, 65.8) BMI: 28.7 (26.3, 32.5) Mortality: 0.34%	I42: Cardiomyopathy (98.0%) I10–I16: Hypertensive diseases (13.4%) I20: Angina pectoris (13.2%) I50: Heart failure (8.54%) I25: Chronic ischemic heart disease (6.12%) I47: Paroxysmal tachycardia (5.78%)
Isolated cardiomyopathy (female)	Cardiomyopathies (94.5%) Osteochondrosis (34.0%) Palpitations (27.5%) Gynecological history (15.3%) Autoimmune thyroiditis (20.3%) Dystrophy (11.3%) Dystonia Disorders (8.92%) Vertebrobasilar Insufficiency (19.2%) Nodular Goiter (20.3%) Unspecified Abortion (17.7%)	N = 2319 69.3% female Age: 46.7 years (33.9, 55.8) BMI: 25.4 (21.9, 29.6) Mortality: 0.12%	I42: Cardiomyopathy (95.2%) E00–E07: Disorders of thyroid gland (10.6%) I10–I16: Hypertensive diseases (9.27%) I49: Other cardiac arrhythmias (6.12%)
Pediatric cardiomyopathy	Birth (86.1%) Cardiac Arrhythmia (94.7%) Pregnancy (85.2%) Childbirth (71.6%) Cardiomyopathies (89.1%) Myocarditis (69.6%) Endocarditis (57.3%) Pericarditis (57.5%) Myocardial dysfunction (54.7%) Viral respiratory infection (66.1%)	N = 745 50.2% female Age: 14.2 years (6.90, 20.5) BMI: 18.8 (15.7, 22.6) Mortality: 0.53%	I42: Cardiomyopathy (69.9%) I50: Heart failure (63.0%) I49: Other cardiac arrhythmias (29.2%) I47: Paroxysmal tachycardia (25.2%) Q20–Q28: Congenital malformations of the circulatory system (24.5%) O99: Other maternal diseases classifiable elsewhere but complicating pregnancy, childbirth and the puerperium (14.7%)

Significance was determined using a one-sided (greater) t-test with Bonferroni correction testing the null hypothesis that the distribution of values of TF-IDF features for a medical entity in cluster i are drawn from the same distribution as the same entity in all other clusters. At right are shown characteristics of heart failure phenotypes, including number of patients, age, sex breakdown, and body mass index (BMI). The “Mortality” statistic denotes the percentage of patients in the cluster that expired within the hospital, as recorded in their EHRs. The “ICD-10” column shows the six most frequent ICD-10 codes and/or groups of codes with more than 5% incidence within the cluster.

**Figure 2:**
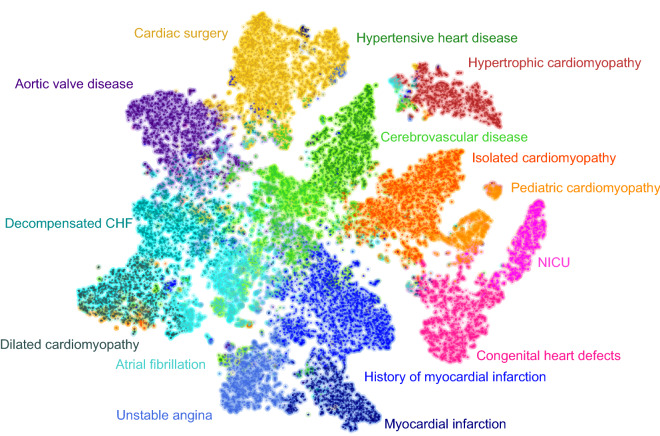
Exemplary 2D visualization of the relative distances between all patients EHRs in the heart failure cohort using t-SNE. Colors show cluster assignment using K-means clustering (K = 15). Each cluster is shown with an interpretable name defining the heart failure phenotype.

Figure 2 shows an exemplary 2D visualization using a t-Distributed Stochastic Neighbor Embedding (t-SNE) based mapping of the HF cohort. Each point represents a single HF patient; the color indicates the cluster assignment for K = 15. From this, we can visualize the relative distance between individual patients in the HF cohort, as well as their respective HF phenotypes.

### Reconstructing a hierarchy of heart failure classification

**Figure 3:**
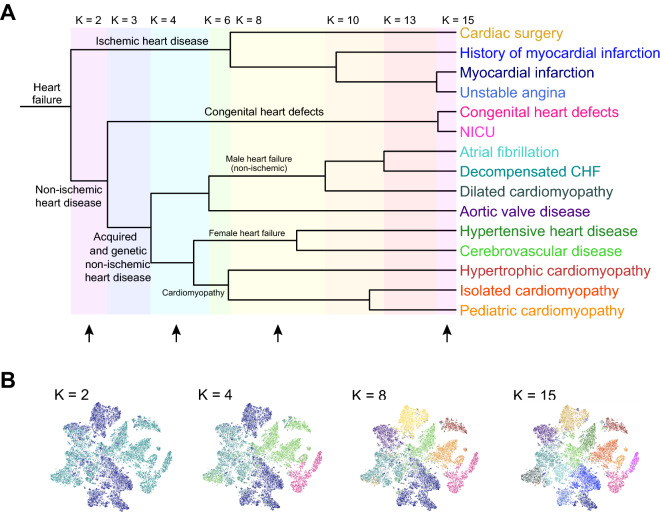
A data-driven hierarchy of HF classification. (**A**) Dendrogram showing hierarchical relationship between cluster phenotypes at different values of K. The dissimilarity metric used to construct distances between clusters at different levels of hierarchy was 1 – *J(C*_*i*_^*K1*^*, C*_*j*_^*K2*^*)*, where *J*(*C*_*i*_^*K1*^*, C*_*j*_^*K2*^) is the Jaccard index between cluster assignment for cluster *i* in for K = K1 (e.g., 15) and cluster j for K = K2 (e.g., 8). Branches are labeled using a clinical interpretation of the hierarchical structure of the clusters (see discussion). (**B**) t-SNE plots showing cluster assignment for K in [2, 4, 8, 15], which are marked with black arrows in (**A**).

Examining Table 2 and Fig. 2, it is apparent that for $K=15$ the population of heart failure patients is grouped into clusters with shared clinical characteristics. Intuitively, we can see that some clusters are more similar to each other than others. To quantify and visualize the natural hierarchy of heart failure within the cohort, we constructed a phenotype dendrogram for $K=15$ (Fig. 3A). The stable clusters used in constructing the dendrogram are $K\epsilon [\mathrm{2,3},\mathrm{4,6},\mathrm{8,10,13}]$ (marked with green corridors in Supplementary Fig. S1). All patients are aggregated at the left side of the dendrogram; each successive branch point shows the value of K at which a cluster splits into two smaller clusters. As $K$ increases, branch points are emphasized with colored highlights; branch points further to the right on the dendrogram represent clusters that are more similar to each other as quantified by their Jaccard index. Thus, we can interpret that at $K=13$ versus $K=15$, *Congenital heart defects* and *NICU* are merged into one cluster, and *Myocardial infarction* and *Unstable angina* are also one cluster. Branches are labeled using a clinical interpretation of the hierarchical structure of the clusters. Figure 3B shows the same t-SNE visualization found in Fig. 2, with cluster assignment colored for values of $K\epsilon [\mathrm{2,4},\mathrm{8,15}]$. This allows us to visualize the same information contained in the dendrogram for selected values of $K$.

From the first branch point in Fig. 3A at $K=2$, we can see the highest level of hierarchy within HF occurs with splitting the HF cohort into groups corresponding to ischemic and non-ischemic heart disease. Next, the non-ischemic heart disease group splits into subgroups that represent congenital vs. acquired and genetic etiologies of heart failure. Finally, at higher levels of $K$, patients within the acquired and genetic non-ischemic heart disease group further fragment into HF subgroups containing atrial fibrillation, dilated cardiomyopathy, aortic valve disease, and decompensated heart failure (which are predominantly comprised of male patients), hypertensive and cerebrovascular disease (predominantly female), and various cardiomyopathies.

### Characterizing properties of discovered phenotypes

Examining the characteristics of each cluster in Table 2 and the dendrogram in Fig. 3, we find well-known interpretable causes and manifestations of heart failure. In the following section, we provide a clinical interpretation of selected clusters for $K=15$. For analysis, the full set of significantly associated concepts (Supplementary Table S2) and descriptive statistics (Table 2) for each cluster was used.

#### Ischemic heart disease

There are four clusters of heart failure patients associated with ischemic heart disease (Fig. 3A, top branch; *Unstable angina*, *History of myocardial infarction*, *Acute myocardial infarction*, and *Cardiac surgery*), the dominant etiology of congestive heart failure^34^. All of these clusters are dominated by males (63.6%, 69.9%, 73.4%, and 79.4%, respectively) and contain patients in their early 60s. Additionally, these clusters all have a similar chronic disease profile based on their ICD-10 codes, and include high prevalence of ischemic heart disease and associated concepts (including coronary heart disease, angina pectoris, myocardial infarction, myocardial ischemia, and stenosis), hypertension, peripheral artery disease, diabetes, stomach disease, and COPD.

Within this group, the two most similar clusters are *Acute myocardial infarction* and *Unstable angina*, both of which contain patients undergoing acute ischemic events. Patients in the *Myocardial infarction* cluster contain patients that experienced an MI (99.1% of patients have the term *myocardial infarction* mentioned in their notes, and 91% of patients have an ICD-10 code for acute myocardial infarction) and have a higher in-hospital mortality (3.01%); we also observe that these patients also have a high prevalence of coronary heart disease (99%), where previous studies have shown the link between coronary heart disease and an increased risk of heart failure after myocardial infarction^35^. Similarly, patients in the *Unstable angina* cluster also have a relatively high rate of ICD-10 codes signifying myocardial infarction (14.5%) but have a higher prevalence of *acute coronary syndrome* (71.0% of patients).

The next branch point includes patients in the cluster named *History of myocardial infarction*. We observe that myocardial infarction is highly associated with this cluster (78.2% of these patients have mentions of *myocardial infarction*) but the EHRs from patients in this cluster have a very low rate of diagnostic codes signifying myocardial infarction. We can interpret this cluster as patients with a history of myocardial infarction and coronary heart disease (mentioned in 99.8% of patients) complicated by heart failure. Finally, the *Cardiac surgery* cluster contains patients who suffer from ischemic heart disease (98.9% of the patients in this cluster contained complaints of *myocardial ischemia*, and 99.5% had complaints of *coronary heart disease*) and underwent cardiac surgery (as evidenced by concepts such as *postpericardiotomy syndrome* in 58.6%, *surgical fistula* in 40.2%, and *wound healing* in 23.7% of patients). Additionally, high prevalence of concepts such as *central venous pressure finding* and *pulmonary artery pressure* indicate the placement of an arterial line, indicating a surgical or other high acuity setting. This interpretation was confirmed via service codes, which reveal that 53.5% of these patients received coronary artery bypass grafting (CABG).

#### Non-ischemic heart disease

##### Etiologies with high morbidity and mortality

We also observe clusters with other well-known non-ischemic etiologies of heart failure; for example *Atrial fibrillation*^36^ and *Heart valve disease*^37,38^. These clusters occur within the same dendrogram branch as *Decompensated CHF* and *Dilated cardiomyopathy*. These clusters share the common characteristic that they are associated with high morbidity and mortality. *Decompensated CHF*, *Dilated cardiomyopathy*, and *Atrial fibrillation* are the only clusters in which mentions of *decompensation* occur significantly more frequently than in other clusters (prevalence of 63.3%, 19.5%, and 24.3% of patients, respectively), which is also reflected in the fact that these clusters group together within the dendrogram; *Decompensated CHF* and *Aortic valve disease* patients have high in-hospital mortality rates of 14.5% and 3.5%, respectively.

*Decompensated CHF* is the cluster with the highest in-hospital mortality rate. In addition to *decompensation*, there are other complaints mentions that show signs of decompensated HF, including *dyspnea* (98.1% of patients) and *acrocyanosis* (43.2%)^39^ as well as poor outcomes such as *respiratory failure* (41.9%), *multiple organ failure* (25.3%), *kidney failure* (24.0%), *cardiac arrest* (27.4%), and *sudden cardiac death* (12.8%). We also observe a high prevalence of *atrial fibrillation* (59.3%) and *chronic kidney diseases* (25.5%), chronic conditions associated with higher risk of and poor outcomes in decompensated HF^39^.

The *Atrial fibrillation* cluster is named for the high prevalence of *atrial fibrillation* (95.7% of patients) and its many variants, including *atrial fibrillation and flutter*, *paroxysmal atrial fibrillation*, *permanent atrial fibrillation, persistent atrial fibrillation*, among others, and other signs of arrhythmia, including *premature ventricular contractions*, *premature cardiac complexes*, *supraventricular arrhythmia*, *tachycardia*, and *bradycardia*. In addition to these classic signs of arrhythmia, these patients also have significant mentions of respiratory problems, including *respiratory insufficiency*, *apnea*, and *obstructive sleep apnea*^40^.

Patients with dilated cardiomyopathy (DCM) were well clustered, with 97.2% of patients in the *Dilated cardiomyopathy* cluster also containing the complaint (as compared to 6.16% of patients outside the cluster), and 93.9% of patients receiving the I42 ICD-10 code for cardiomyopathy. These patients also exhibited typical complaints for DCM, including *dyspnea* (90.6% of patients); *edema* (67.0%) and *swelling* (41.2%); valve insufficiencies such as *mitral valve insufficiency* (82.8%) and *tricuspid valve insufficiency* (76.0%); arrhythmias, including *ventricular tachycardia* (37.7%), *premature ventricular contractions* (67.3%), *interventricular desynchrony* (18.9%), and *atrial fibrillation* (39.8%), among others; and *pulmonary embolism* (17.2%)^41^. Although incidence of DCM is typically biased towards men^42^, there is a disproportionally low proportion of females in the cohort (21.6%). This was hypothesized to be due to the Russian origin of the dataset, where rates of alcohol consumption in males and corresponding alcoholic cardiomyopathy are high^43,44^. Subsequent analysis identified EHR templates documenting lifestyle risk factors; within the DCM group, 33.12% of the patients had documentation of alcohol consumption as a bad habit, compared to 1.7% of patients outside of the DCM cluster. Thus, this cluster can also be interpreted as *Alcoholic cardiomyopathy*.

The *Heart valve disease* cluster contains patients with high prevalence of heart valve disease, including *aortic valve disease*, *aortic valve stenosis*, *heart valve disease*, *mitral valve insufficiency*, *tricuspid valve insufficiency*, and *chronic rheumatic heart disease*, among others. Findings of *central venous pressure finding*, *cardiac index*, and *cardiac activity*, and *wound healing*, as well as complaints such as *postpericardiotomy syndrome*, show that these patients had conditions serious enough to warrant surgeries and possible ICU admissions, and may explain the high mortality rate. Analysis of service codes revealed that the *Heart valve disease* cluster experienced the second highest rates of surgery of any cluster (after *Cardiac surgery*), where 34.9% of patients received any form of cardiac surgery, and 22.4% received valvuloplasty or prosthetic valves.

##### Female heart failure

Within Fig. 3A, there is a branch containing clusters labeled *Hypertensive heart disease* and *Cerebrovascular disease*. In these clusters, we observe other well-known comorbidities and etiologies of heart failure including hypertension^45,46^ and vascular disease (including cerebrovascular disease and stroke) concomitant with conditions such as diabetes and chronic kidney disease (CKD)^47^. Both clusters have a high percentage of female patients (72.3% in *Hypertensive heart disease* and 58.2% in *Cerebrovascular disease*).

Patients within the *Hypertensive heart disease* cluster have the highest BMI out of any cluster, with a median value of 30.1, as well as text mentions of complaints such as *hyperlipidemia* (43.1% of patients) and *obesity* (46.4% of patients). The high fraction of females is supported by literature, as progression of hypertension as the primary etiology of heart failure is up to 50% more common in women than men^45^. Patients within the *Cerebrovascular disease* contain the oldest patients (median age 67.3); within this cluster complaints include mentions of vascular conditions such as *vascular diseases* (29.8% of patients), *cerebral atherosclerosis* (40.1%), *peripheral arterial disease* (13.7%), *ischemic stroke* (14.3%), *cerebral infarction* (10.0%), and *cerebrovascular accident* (65.5%); neurological symptoms such as *encephalopathies* (67.2%), *nystagmus* (34.7%), and *dysarthria* (52.5%); common complications of stroke such as *hemiparesis* (12.9%); and chronic conditions including *diabetes mellitus* (46.1%), *diabetic polyneuropathies* (21.0%), *chronic kidney diseases* (17.8%), and *diabetic nephropathy* (14.4%). Together, the predominance of overweight or obese female patients with a heavy burden of comorbid conditions are consistent with characteristics of HFpEF^48–50^.

### Validating and discovering relations between HF patient complaints

From the previous sections, the proposed clustering framework validates well-known findings. To further quantify these interpretations, we used the concept association score ${a}_{ij}$ to quantify how frequently concepts co-occur for concept pairs where both concepts are significantly associated with the same cluster, as well as for concept pairs that are significantly associated with different clusters. Our analysis shows that the concept association scores within clusters are higher than those between clusters (mean 0.3016 vs. 0.1143, $p=2.00\times 10^{-168}$, t-test). Furthermore, these results are replicated in PubMed, where we observe the same pattern (mean 0.0341 vs. 0.0054, $p=1.58\times 10^{-23}$, t-test).

Table 3 shows concept association scores for selected concept pairs. In several cases, we observe that the association score is lower in PubMed than in our data. This suggests that our method can be an approach to gather evidence for or discover lesser known associations between medical concepts. One such example is the co-occurrence of decompensated heart failure and lymphadenopathy. The association of hilar and mediastinal lymphadenopathy as a finding of decompensated heart failure has been established but not well studied^51–53^; as of 2016, the largest study on the association between acute heart failure and lymphadenopathy contained a cohort of 215 patients (original cohort of 500 HF patients, with 285 excluded for lack of CT scans or possible confounding diagnoses that can cause lymphadenopathy), of which 68% exhibited CT signs of lymphadenopathy^54^. Within the cluster of “Decompensated CHF”, 1368 patients contained complaints mentions of *decompensation*, with 486 of these patients (35.5%) containing mentions of *lymphadenopathy*, *mediastinal lymphadenopathy*, or *hilar lymphadenopathy* (see Supplementary Table S2), all of which were significantly associated with the cluster.

**Table 3:** Exemplary association scores between pairs of medical concepts that co-occur within cluster phenotypes from Table 2.

Cluster name	Term 1	Term 2	Association score: HF cohort	Association score: PubMed
Atrial fibrillation	Atrial fibrillation	Cardiac arrhythmia	0.356738	0.238322
Atrial fibrillation	Atrial fibrillation	Varicosity	0.428386	0.000394
Atrial fibrillation	Mitral valve insufficiency	Subclinical hypothyroidism	0.130795	0
Decompensated CHF	Pulmonary embolism	Thrombus	0.301952	0.150213
Decompensated CHF	Decompensation	Lymphadenopathy	0.246885	0.000425
Decompensated CHF	Lymphadenopathy	Mitral valve insufficiency	0.165153	0.000082
Hypertensive heart disease	Heart diseases	Heart failure	0.292404	0.154376
Hypertensive heart disease	Hyperlipidemia	Obesity	0.226397	0.026089
Hypertensive heart disease	Obesity	Vertebrobasilar insufficiency	0.134699	0.000024
Isolated cardiomyopathy	Cardiomyopathies	Myocarditis	0.222379	0.154172
Isolated cardiomyopathy	Autoimmune thyroiditis	Cardiomyopathies	0.125604	0.000934
Isolated cardiomyopathy	Cardiomyopathies	Osteochondrosis	0.146162	0.000031

Comparable association scores within the HF cohort and the scientific literature (PubMed) indicate that co-occurrences are already known. Significantly higher association scores in the HF cohort indicate potentially novel associations.

This result shows the power of RWD analysis to provide additional support for furthering our understanding of HF and its pathophysiology, which may aid in differential diagnosis and improved quality of care (e.g., reduction in unnecessary lymph node biopsies).

## Discussion

In this study, we presented a novel and data-driven approach to constructing HF phenotypes based on the real-world disease manifestation found in the unstructured clinical notes of a large EHR database. These phenotypes were discovered by utilizing an NLP-based information extraction and unsupervised clustering approach to group HF patients into subcohorts. For $K=15$, we interpreted each subcohort by employing a statistical testing methodology in which we found medical concepts and complaints that are overrepresented in each group. We also characterized descriptive statistics of each subcohort, including demographic information such as age and sex, BMI, in hospital mortality rates, and most frequent diagnosis codes. Additionally, we used clustering at different value of *K*, which revealed the hierarchy of HF phenotypes. Finally, after finding the significant concepts and descriptive statistics for each cluster, we provided a medical analysis and find that subcohorts correspond to clinically meaningful etiologies and endpoints of heart failure.

Clinical notes are a rich source of patient information but are underutilized due to the major challenges involved in extracting and normalizing medical concepts found in unstructured free text. Here we found that hierarchical, data-driven phenotypes for heart failure could be constructed solely by clustering of complaints (disease and symptom mentions) extracted from unstructured notes. The resulting HF phenotypes are clinically informative with respective to comorbid conditions, symptoms, and other complaints that may be missing from traditional HF classifications, providing a more complete picture of a patient’s disease state. These results are illuminating with regard to both the etiology and severity of HF across the cohort and provide a snapshot of disease characteristics across a large population.

In contrast to top-down approaches that use predefined criteria to classify HF disease states, this unsupervised approach using unstructured clinical notes offers the ability to uncover HF patient subgroups across multiple scales of medical concept granularity based on real-world data. Such an approach is data-driven and flexible; by discovering subcohorts of patients based on the similarity of their complaints, there is no need to specify complex inclusion/exclusion criteria a priori, but rather allow for dominant patterns to be discovered from the data itself.

### Potential applications

Development of more effective methods for understanding heart failure in its various clinical manifestations, its symptoms, and their management is vital to improving treatment strategies and ultimately the quality of life of HF patients. The ability of our approach to automatically reveal patterns of real-world disease manifestations can aid in understanding complex syndromes like HF and the phenotypic heterogeneity in its patient populations. Importantly, we demonstrate that our methodology is able to produce an automated and scalable understanding of a large population of HF patients using a health system’s routinely collected clinical data, which can serve as a foundation for practice-based medicine in which real-world insights relevant to a patient can be generated and provided to a clinician at the point of care^55^. For example, it is challenging for providers to understand which care regimens should be used in each patient subpopulation, particularly when dealing with older, chronic disease patients with multiple comorbidities. Phenotype clusters built from retrospective data can be a powerful tool to drive better treatment decisions; through analysis of which treatments have been successful for a given cluster in the past, providers may gain insight into which care regimens would have the best chance of success for new patients that map to existing clusters, thus enabling cluster-specific personalization of care.

Analogously, this technique can be applied on large cross-provider patient populations for epidemiology or health economics and outcomes research. Having phenotypes that more accurately reflect disease manifestations in real patient populations can improve the precision of disease burden assessments, which in turn can help healthcare practitioners or policy makers understand likely outcomes for large segments of the population and better perform resource allocation.

Finally, the patient representations built using this method present a unique opportunity to extract insights that can be shared between hospitals, because they extract high-level complaints without using patient identifiers. Additionally, because the method utilizes a clinical NLP system that extracts language-independent medical concepts from clinical text, such an approach can allow for scalable comparison of patient populations across different regions and languages without building word or term mappings to standard controlled terminologies, which is often prohibitively time-consuming in practice.

### Limitations and future directions

Although a relatively large number of patient records were used in this study (n = 25,952), it remains to be determined whether the HF phenotypes reported here will remain comprehensive across larger patient populations and geographies and is a future direction of study. Additionally, in this study results were generated using complaints extracted from clinical text in the EHR without using (1) any structured data or (2) unstructured data corresponding to clinical interventions (e.g., medications, procedures) or numerical value extraction for lab and imaging measurements. Future avenues of research can explore utilizing structured sources of information in the EHR (e.g., diagnosis codes, labs) to enrich or further inform cluster phenotypes. Additionally, our general approach can be supplemented with other healthcare data sources, including other regularly collected information (e.g., administrative data or claims) or data sources used in precision medicine, if they are available (e.g., omics data). While the current approach identifies HF phenotypes by clustering on aggregated complaints extracted from entire patient timelines, an important direction for future research is to (1) analyze the progression of HF patients and the evolution of their disease state over time, and (2) study the interplay between phenotype, clinical interventions, and ultimately patient outcomes. Finally, in the current study we demonstrate the viability of data-driven phenotypes in heart failure, but the approach is condition-agnostic and can be easily applied to other diseases areas in the future.

## Supplementary information

Supplementary Information.

Supplementary Table S2.

## Data Availability

The datasets generated during this study are available from the corresponding author on reasonable request.

## References

[1] Yancy CW (2013). ACCF/AHA guideline for the management of heart failure: a report of the American college of cardiology foundation/american heart association task force on practice guidelines. J. Am. Coll. Cardiol..

[2] Warriner D, Sheridan P, Lawford P (2015). Heart failure: not a single organ disease but a multisystem syndrome. Br. J. Hosp. Med..

[3] Mcmurray JJV (2012). ESC guidelines for the diagnosis and treatment of acute and chronic heart failure 2012. Eur. J. Heart Fail..

[4] Desai RJ (2019). Comparative effectiveness of generic and brand-name medication use: a database study of us health insurance claims. PLoS Med..

[5] Ponikowski P (2016). ESC guidelines for the diagnosis and treatment of acute and chronic heart failure. Eur. Heart J..

[6] Yancy CW (2017). ACC/AHA/HFSA focused update of the 2013 ACCF/AHA guideline for the management of heart failure: a report of the American college of cardiology/american heart association task force on clinical practice guidelines and the heart failure society of America. J. Am. Coll. Cardiol..

[7] Nomenclature and criteria for diagnosis of diseases of the heart and great vessels. *Ann. Intern. Med.* (1974)

[8] De Keulenaer GW, Brutsaert DL (2007). Systolic and diastolic heart failure: Different phenotypes of the same disease?. Eur. J. Heart Fail..

[9] Neill DB, Heinz HJ (2015). Subtyping: What it is and its role in precision medicine. IEEE Intell. Syst..

[10] Boland MR, Hripcsak G, Shen Y, Chung WK, Weng C (2013). Defining a comprehensive verotype using electronic health records for personalized medicine. J. Am. Med. Inform. Assoc..

[11] Austin PC, Tu JV, Ho JE, Levy D, Lee DS (2013). Using methods from the data-mining and machine-learning literature for disease classification and prediction: a case study examining classification of heart failure subtypes. J. Clin. Epidemiol..

[12] Alonso-Betanzos A, Bolón-Canedo V, Heyndrickx GR, Kerkhof PLM (2015). Exploring guidelines for classification of major heart failure subtypes by using machine learning. Clin. Med. Insights Cardiol..

[13] Koleck TA, Dreisbach C, Bourne PE, Bakken S (2019). Natural language processing of symptoms documented in free-text narratives of electronic health records: a systematic review. J. Am. Med. Inform. Assoc..

[14] Nagamine, T., Gillette, B., Makarov, A., Kahoun, J. & Saxena, M. Estimating the burden of major diseases in Russia from electronic health records using a multilingual clinical natural language processing system (in preparation).

[15] Senni M (2014). New strategies for heart failure with preserved ejection fraction: the importance of targeted therapies for heart failure phenotypes. Eur. Heart J..

[16] Burgel PR (2010). Clinical COPD phenotypes: a novel approach using principal component and cluster analyses. Eur. Respir. J..

[17] Garcia-Aymerich J (2011). Identification and prospective validation of clinically relevant chronic obstructive pulmonary disease (COPD) subtypes. Thorax.

[18] Haldar P (2008). Cluster analysis and clinical asthma phenotypes. Am. J. Respir. Crit. Care Med..

[19] Gligorijevic D, Stojanovic J, Obradovic Z (2016). Disease types discovery from a large database of inpatient records: a sepsis study. Methods.

[20] Richette P, Clerson P, Périssin L, Flipo RM, Bardin T (2015). Revisiting comorbidities in gout: a cluster analysis. Ann. Rheum. Dis..

[21] Fereshtehnejad SM, Romenets SR, Anang JBM, Latreille V, Gagnon JF, Postuma RB (2015). New clinical subtypes of Parkinson disease and their longitudinal progression a prospective cohort comparison with other phenotypes. JAMA Neurol..

[22] Ahmad T (2014). Clinical implications of chronic heart failure phenotypes defined by cluster analysis. J. Am. Coll. Cardiol..

[23] Obokata M, Reddy YNV, Pislaru SV, Melenovsky V, Borlaug BA (2017). Evidence supporting the existence of a distinct obese phenotype of heart failure with preserved ejection fraction. Circulation.

[24] Horiuchi Y (2018). Identifying novel phenotypes of acute heart failure using cluster analysis of clinical variables. Int. J. Cardiol..

[25] Vazquez Guillamet R, Ursu O, Iwamoto G, Moseley PL, Oprea T (2018). Chronic obstructive pulmonary disease phenotypes using cluster analysis of electronic medical records”. Health Inform. J..

[26] Nowbar AN, Gitto M, Howard JP, Francis DP, Al-Lamee R (2019). Mortality from ischemic heart disease. Circ. Cardiovasc. Qual. Outcomes.

[27] Murphy A (2018). Ischaemic heart disease in the former Soviet Union 1990–2015 according to the Global Burden of Disease 2015 Study. Heart.

[28] Starodubov VI (2018). The burden of disease in Russia from 1980 to 2016: A systematic analysis for the global burden of disease study 2016. Lancet.

[29] Bишнeвcкий AГ, Aндpeeв EM, Tимoнин CA (2016). Cмepтнocть oт бoлeзнeй cиcтeмы кpoвooбpaщeния и пpoдoлжитeльнocть жизни в Poccии. Дeмoгpaфичecкoe oбoзpeниe.

[30] Bodenreider O (2004). The unified medical language system (UMLS): Integrating biomedical terminology. Nucleic Acids Res..

[31] SNOMED, C.T. https://www.nlm.nih.gov/healthit/snomedct/index.html.

[32] Ramos, J. Using TF-IDF to determine word relevance in document queries (2003).

[33] Lloyd SP (1982). Least squares quantization in PCM. IEEE Trans. Inf. Theory.

[34] Remme WJ (2000). Overview of the relationship between ischemia and congestive heart failure. Clin. Cardiol..

[35] Gerber Y (2016). Atherosclerotic burden and heart failure after myocardial infarction. JAMA Cardiol..

[36] Anter E, Jessup M, Callans DJ (2009). Atrial fibrillation and heart failure: Treatment considerations for a dual epidemic. Circulation.

[37] Nishimura RA (2002). Aortic valve disease. Circulation.

[38] Kamperidis V, Delgado V, Van Mieghem NM, Kappetein AP, Leon MB, Bax JJ (2016). Diagnosis and management of aortic valve stenosis in patients with heart failure. Eur. J. Heart Fail..

[39] Mangini S, Vieira Pires P, Goulart F, Braga M, Bacal F (2013). Decompensated heart failure. Einstein (Sao Paulo).

[40] Marulanda-Londoño E, Chaturvedi S (2017). The interplay between obstructive sleep apnea and atrial fibrillation. Front. Neurol..

[41] Lynn J, Eries J, Rey J, Towbin A (2010). Dilated cardiomyopathy. Lancet.

[42] Fairweather DL, Cooper LT, Blauwet LA (2013). Sex and gender differences in myocarditis and dilated cardiomyopathy. Curr. Probl. Cardiol..

[43] Leon DA, Shkolnikov VM, McKee M, Kiryanov N, Andreev E (2010). Alcohol increases circulatory disease mortality in Russia: Acute and chronic effects or misattribution of cause?. Int. J. Epidemiol..

[44] Keenan K, Saburova L, Bobrova N, Elbourne D, Ashwin S, Leon DA (2015). Social factors influencing Russian male alcohol use over the life course: a qualitative study investigating age based social norms, masculinity, and workplace context. PLoS ONE.

[45] Messerli FH, Rimoldi SF, Bangalore S (2017). The transition from hypertension to heart failure contemporary update. JACC Heart Fail..

[46] Rodeheffer RJ (2011). Hypertension and heart failure: the allhat imperative. Circulation.

[47] Dokken BB (2008). The pathophysiology of cardiovascular disease and diabetes: beyond blood pressure and lipids. Diabetes Spectr..

[48] Lekavich CL, Barksdale DJ, Neelon V, Wu JR (2015). Heart failure preserved ejection fraction (HFpEF): an integrated and strategic review. Heart Fail. Rev..

[49] Oktay AA, Rich JD, Shah SJ (2013). The emerging epidemic of heart failure with preserved ejection fraction. Curr. Heart Fail. Rep..

[50] Kitzman DW, Shah SJ (2016). The HFpEF obesity phenotype. J. Am. Coll. Cardiol..

[51] Slanetz PJ, Truong M, Shepard JA, Trotman-Dickenson B, Drucker E, McLoud TC (1998). Mediastinal lymphadenopathy and hazy mediastinal fat: new CT findings of congestive heart failure. Am. J. Roentgenol..

[52] Pastis NJ (2011). Mediastinal lymphadenopathy in patients undergoing cardiac transplant evaluation. Chest.

[53] Nin CS (2016). Thoracic lymphadenopathy in benign diseases: A state of the art review. Respir. Med..

[54] Shweihat YR (2016). Congestive adenopathy. J. Bronchol. Interv. Pulmonol..

[55] Hemingway H (2018). Big data from electronic health records for early and late translational cardiovascular research: challenges and potential. Eur. Heart J..

