# Supplementary material for: Multiscale classification of heart failure phenotypes by unsupervised clustering of unstructured electronic medical record data
Source: Sci Rep. 2020 Dec 7;10:21340. doi: 10.1038/s41598-020-77286-6 (PMC7721729; doi:10.1038/s41598-020-77286-6)
Supplement: Supplementary file 1 — Supplementary Information. [file 41598_2020_77286_MOESM1_ESM.docx]

# Multiscale Classification of Heart Failure Phenotypes by Unsupervised Clustering of Unstructured Electronic Medical Record Data

Tasha Nagamine^†^, Brian Gillette^‡¶^, Alexey Pakhomov^†^, John Kahoun^†§^, Hannah Mayer^#^, Rolf Burghaus^#^, Jörg Lippert^#^, Mayur Saxena^†*^

† Droice Research, New York, NY, United States

‡ Department of Surgery, NYU Winthrop Hospital, Mineola, NY, United States

¶ Department of Foundations of Medicine, NYU Long Island School of Medicine, Mineola, NY, United States

§ Clinical Informatics, CityMD, New York, NY, United States
# Clinical Pharmacometrics, Bayer AG, Wuppertal, Germany

* Correspondence to [mayur@droicelabs.com](mailto:mayur@droicelabs.com)

## Supplementary Materials and Methods

#### TF-IDF Implementation

The vector of counts of complaints of each patient was transformed using the term frequency-inverse document frequency (TF-IDF) approach. Copy and pasting is common in clinical text and becomes even more prevalent in patients with longer records. To reduce the contribution of repeated text chunks and normalize term counts over patients with different timeline lengths, we utilized a logarithmic term frequency, followed by an L2 normalization of each patient vector.

The relative frequency of complaints in the dataset follows a power law distribution. To limit the dimensionality of the representation and to exclude rare terms, we restricted the complaints vocabulary $\boldsymbol{V}$ to 99% the most frequent complaints, which reduced the vocabulary size from 9,375 to 1,276. Vectorizing the entire heart failure cohort results in a two-dimensional matrix of the form $\boldsymbol{P}=(p_{i,j})\epsilon\mathbb{R}^{N\times V}$. Here, **N** denotes the number of patients in the cohort.

#### Inferring Phenotypes from Clusters

To interpret clusters of patients discovered via K-means clustering, we used statistical testing to find complaints that were significantly overrepresented within the cluster as compared to the rest of the heart failure population. Doing so allows us to determine the distinguishing medical concepts, or features, of each cluster. More specifically, for each cluster $i$ we break the patient matrix $\boldsymbol{P}$ into two submatrices, $\boldsymbol{P}_{i}$ and $\boldsymbol{P}_{j}$, where $\boldsymbol{P}_{i}$ contains the data points of all patients from cluster $i$ ($k=i$) and $\boldsymbol{P}_{j}$, contains the data points from all other clusters ($k\neq i$). For each feature $f$, we then use a one-sided t-test to test the null hypothesis that the mean of the TF-IDF features in $\boldsymbol{P}_{i}^{f}$ are equal to $\boldsymbol{P}_{j}^{f}.$ Rejection of the null hypothesis for feature $f$ means that this complaint is overrepresented in cluster $i$ and can be interpreted as a distinguishing characteristic of the cluster. Performing this test for each complaint within a cluster yields a vocabulary of significantly associated complaints $\boldsymbol{V}_{s}$. We employed Bonferroni correction for multiple comparisons. Samples were tested to confirm to be normally distributed.

#### Clustering Model Evaluation

We hypothesized that clustering with different K values (numbers of clusters) would group HF patients by different levels of disease hierarchy (i.e., small values of K would result in cohorts grouped by broad disease features, whereas a large values of K would results in cohorts grouped by more granular disease features). To determine which values of K yield stable clusters for the HF patent dataset, we quantified cluster stability for each value of K with a bootstrapping strategy (1). For values of K that represent true clusters in the underlying dataset, these clusters should be re-identifiable in randomly subsampled portions of the original dataset.

First, for $K\epsilon[2,3,\ldots,30]$, we clustered patients via K-means clustering to obtain reference clustering results $[C_{K=1}^{\mathrm{ref}}, C_{K=2}^{\mathrm{ref}}, ..., C_{K=30}^{\mathrm{ref}}]$. Then for each reference clustering result for each value of K, we utilized a cluster bootstrapping strategy in which we 1) subsampled a fixed fraction of the original dataset $f_{d}$then 2) clustered again to generate a bootstrapped clustering result $C_{K=k}^{f_{d}}$. We repeated the bootstrapping for varying fractions of data $f_{d}\epsilon[0.5,0.25,...,0.0078125]$ to investigate whether the size of the available patient cohort would limit the level of accuracy and robustness of the cluster identification. This procedure was repeated 1,000 times for each value of K and each fraction of data.

To evaluate the stability of clustering results, we compared the similarity of each bootstrapped clustering result to the corresponding reference clustering at each value of $K$. The Jaccard index characterizes how robust the grouping of patients within a cluster is to changes in the specific population of patients through random subsampling of the data set. Similarity was thus calculated using the Jaccard index across the reference and bootstrapped clustering results:

$$J(C_{K=k}^{\mathrm{ref}}, C_{K=k}^{f_{d}})=\frac{1}{N}\sum_{i=1}^{k} \sum_{j=1}^{k} n_{ij} \cdot J_{ij}$$

Here $N$ denotes the number of datapoints used in both clustering models, $n_{ij}$ is the number of datapoints present in cluster $i$ from reference clustering $C_{K=k}^{\mathrm{ref}}$ and cluster $j$ from bootstrapped clustering $C_{K=k}^{f_{d}}$. $J_{ij}$ is the Jaccard index between in clusters $i$ and $j$:

$$J_{ij} = \frac{\left| c_{i}^{\mathrm{ref}} \bigcap c_{j}^{f_{d}} \right|}{\left| c_{i}^{\mathrm{ref}} \bigcup c_{j}^{f_{d}} \right|}$$

For each value of K and each fraction of data, this analysis yields a distribution of 1000 values of $J$, which can be used to quantitatively compare the stability of clustering across different values of K. In this formulation, a value of $J=1$ means that the data has been partitioned identically in the reference and bootstrapped clustering (perfect stability). As less data is sampled for bootstrapping (lower values of $f_{d}$), we can empirically test the amount of data at which the clustering result breaks down.

To test the stability of the medical concepts characterizing the different patient clusters, we utilized a *semantic similarity* measure based on a modification of the Jaccard index. S*emantic similarity S* quantifies the similarity of the significant complaints associated with each cluster between the reference and bootstrapped clustering results:

$$S(C_{K=k}^{\mathrm{ref}}, C_{K=k}^{f_{d}})=\frac{1}{N}\sum_{i=1}^{k} \sum_{j=1}^{k} n_{ij} \cdot S_{ij}$$

As before, $N$ denotes the number of datapoints used in both clustering models, $n_{ij}$ is the number of datapoints present in cluster $i$ from reference clustering $C_{K=k}^{\mathrm{ref}}$ and cluster $j$ from bootstrapped clustering $C_{K=k}^{f_{d}}$. $S_{ij}$ is the semantic similarity index between in clusters $i$ and $j$, and quantifies the overlap of the significantly associated complaints vocabularies between the two clusters:

$$S_{ij} = \frac{\left| v_{s,i}^{\mathrm{ref}} \bigcap v_{s,j}^{f_{d}} \right|}{\left| v_{s,j}^{f_{d}} \right|}$$

As with the Jaccard index bootstrapping, we repeated the bootstrapping for varying fractions of data $f_{d}\epsilon[0.5,0.25,...,0.0078125]$. Robust identification of local maxima in $J_{ij}$ and $S_{ij}$ across increasingly diminishing fractions of the data provides the basis for selection of stable clusters.

#### Selection of K and Inferring Phenotype Hierarchies

In this analysis, we considered local maxima when sampling 50% of data ($f_{d}=0.5$) resulting in $m$ values of $K\epsilon[k_{max}^{1},k_{max}^{2},\ldots,k_{max}^{m}]$. After choosing to analyze a set of clusters satisfying both local maximum criteria at a given value of $K$, which we denote $m^{*}$, we aimed to visualize the hierarchical structure of the clustering result. Doing so allows us to understand which phenotypes are more similar to each other and thus frame the results at various levels of hierarchy. To do so, we computed the Jaccard index between the clustering results $C_{max}^{m*}$ and the clustering result for all local maxima for values of $K$ less than $m^{*}$ $C_{max}^{m}$, $m \epsilon[m^{*}-1,m^{*}-2,\ldots,1]$. This results in a Jaccard similarity matrix of size $[m^{*} \times m]$; we then computed the pairwise distance between each cluster in $C_{max}^{m*}$. We repeated this for each value of $m$, computed the average, then use the resulting combined distance matrix to create a dendrogram using hierarchical clustering (complete linkage). The resulting phenotype dendrogram allows us to understand the hierarchical relationship between clusters at different values of $K$ and provides a *phylogenetic tree of complaints and symptoms*.

### Quantifying associations between medical concepts

Finally, we performed an analysis to quantify the co-occurrence rate of important (significantly overrepresented) concepts associated with each cluster. To quantify the co-occurrence of concepts associated with each phenotype, we considered the top 10 most significantly associated (smallest p-value) concepts in each cluster. Thus, for a given value of $K$, we consider $10K$ concepts; we then calculate a *concept association score* $a_{ij}$ between a pair concepts $c_{i}$ and $c_{j}$, using the Jaccard index:

$$a_{ij} = \frac{N_{ij}}{N_{i}+N_{j}-N_{ij}}$$

Here, $N_{ij}$ denotes the number of patients in which $c_{i}$ and $c_{j}$ are both mentioned, while $N_{i}$ and $N_{j}$ denote the number of patients in which $c_{i}$ and $c_{j}$ occur, respectively.

To quantitatively compare associations in published literature, we also computed the same pairwise scores using PubMed queries. For PubMed, $N_{ij}$ denotes the number of articles in which $c_{i}$ and $c_{j}$ are both mentioned, while $N_{i}$ and $N_{j}$ denote the number of articles in which $c_{i}$ and $c_{j}$ occur, respectively.

#### Statistical Package Versions

TF-IDF vectorization, K-means clustering, and t-SNE were implemented using Python’s Scikit-learn (0.19.1). Statistical tests were implemented using Scipy (1.3.0).

## Supplementary Results

### Characterizing Properties of Discovered Phenotypes

#### Non-Ischemic Heart Disease

##### Congenital Heart Disease

A branch point in Fig. 3A ($K=3$) occurs within non-ischemic heart disease and separates neonates with congenital heart disease from the rest of the non-ischemic heart disease groups. These two clusters contain infants (*Neonatal ICU* and *Congenital Heart Defects*, median age of 1.33 and 0.08 years, respectively). Within each of these clusters over 98% of patients are coded with the ICD-10 group Q20-Q28 (congenital malformations of the circulatory system); this and the young age of patients within these groups points to a congenital origin of HF, a common etiology of heart failure in younger patients (2). This conclusion is supported by the significant concepts associated with these clusters, including terms such as *congenital heart defects*, *congenital heart disease*, *congenital abnormality*, *atrial septal defects*, *ventricular septal defects*, and *atrioventricular septal defects*.

From Fig. 3A, we can see that patients within these two phenotypes cluster together even at $K=13$ and only break into two groups at $K=15$. Although these clusters share the same etiology of heart failure, the patients in *Neonatal ICU* have a much more severe condition than those in *Congenital Heart Defects*. In addition to containing mentions of congenital heart disease, the patients in *Neonatal ICU* also contain mentions of medical concepts such as *surgical wound* (found in 76.7% of patients), *hypotension* (80.0%), *respiratory failure* (71.2%), *acute kidney failure* (41.9%), *air embolism* (62.2%), *oxygen saturation below reference range* (46.8%), and *ventricular hemorrhage* (37.1%); the severity of their condition is reflected in an in-hospital mortality rate of 16.6%. Additionally, 57.% of these patients had mentions of *infants, premature* and 57% had mentions of *premature birth*, a condition associated with HF in the young (3).

##### Cardiomyopathy

We observed causes of heart failure corresponding to cardiomyopathy, including hypertrophic cardiomyopathy (4,5) as well as unspecified cardiomyopathies. These are distributed within four clusters where a majority of the patients have several types of cardiomyopathy according to both significant concepts and ICD-10 codes: *Hypertrophic cardiomyopathy* (HCM), *Isolated cardiomyopathy (adult)*, and *Pediatric cardiomyopathy*. Aside from the neonates, these three groups have some of the youngest patients (median age of 55, 45, and 11 respectively).

Patients with HCM were well-clustered within the HF cohort; the complaint *Hypertrophic cardiomyopathy* is mentioned in the clinical text 100% of the patients of the eponymous cluster (and is only present in 2.58% of patients outside the cluster), and 98.0% of these patients received the I42 ICD-10 code for cardiomyopathy. These patients also exhibit complaints that are typical complications of HCM, including *syncope* (16.9% of patients), *heart murmur* (34.0%), and *mitral valve insufficiency* (77.1%) (6). Interestingly, although most studies show a bias toward men of up to 60% in HCM cohorts, our study contains a relatively gender-balanced cohort (53.9% female), which is consistent with the autosomal dominant inheritance pattern of this disorder (7).

The *Isolated cardiomyopathy* cluster is predominantly female (69.3%) and is characterized by high rates of thyroid disorders, including mentions of *autoimmune thyroiditis* (20.3% of patients), *nodular goiter* (20.3%), *hypothyroidism* (12.5%), *thyrotoxicosis* (7.50%), *toxic diffuse goiter* (4.13%), and *Grave’s disease* (3.01%). After cardiomyopathies, the next most common diagnostic codes in this group were associated with thyroid disease. This is consistent with the fact that thyroid disorders are more common in females (8) and can serve as a driver of heart disease, including cardiomyopathy and heart failure (9).

The *Pediatric cardiomyopathy* cluster contains patients with a median age of 11.7 (4.39, 17.7 interquartile range). Based on the significant concepts associated with this group, this cluster seems to contain a mix of the youngest heart failure patients (excluding neonates with congenital heart disease). First, 69.6% of patients within this group had mentions of *myocarditis* mentioned in their notes (with *endocarditis* and *pericarditis* also significantly associated), which is known to cause cardiomyopathy (10) and lead to heart failure in children (11). Although very likely originating from a different etiology, this cluster also appears to contain patients with peripartum cardiomyopathy, as evidenced by significant concepts such as *pregnancy*, *childbirth*, *primigravida*, *premature birth*, *breast feeding*, *preeclampsia*, *fetal movement*, and *duration of gestation*, where previous studies have outlined the close relationship between pregnancy, myocarditis, and cardiomyopathy (12).

Finally, it should be noted that out of all the cardiomyopathy clusters, the DCM cluster was the only one that was not grouped with the rest of cardiomyopathy clusters in the phenotype dendrogram but was instead grouped closer to *Decompensated CHF* and *Atrial fibrillation*. As discussed above, this is likely due to the fact that the DCM cluster exhibits several clinical characteristics shared with to *Decompensated CHF* and *Atrial fibrillation*, including a predominantly male cohort and high rates of mentions of *decompensation* and *atrial fibrillation*.

## Supplementary References

1. Hennig C. Cluster-wise assessment of cluster stability. Comput Stat Data Anal. 2007 Sep 15;52(1):258–71.

2. Oechslin EN, Harrison DA, Connelly MS, Webb GD, Siu SC. Mode of Death in Adults With Congenital Heart Disease. Vol. 86, Excerpta Medica, Inc. 2000.

3. Carr H, Cnattingius S, Granath F, Ludvigsson JF, Bonamy A-KE. Preterm Birth and Risk of Heart Failure Up to Early Adulthood. 2017.

4. Lynn J, Eries J, Rey J, Towbin A. Dilated cardiomyopathy [Internet]. Vol. 375, www.thelancet.com. 2010. Available from: www.thelancet.com

5. Towbin JA, Bowles KR, Bowles NE. Etiologies of cardiomyopathy and heart failure. Nat Med [Internet]. 1999;5(3):266–7. Available from: https://doi.org/10.1038/6474

6. Maron BJ. Hypertrophic Cardiomyopathy A Systematic Review [Internet]. Available from: https://jamanetwork.com/

7. Siontis KC, Ommen SR, Geske JB. Sex, Survival, and Cardiomyopathy: Differences Between Men and Women With Hypertrophic Cardiomyopathy. Vol. 8, Journal of the American Heart Association. NLM (Medline); 2019. p. e014448.

8. Mulder JE. THYROID DISEASE IN WOMEN. Med Clin North Am [Internet]. 1998;82(1):103–25. Available from: http://www.sciencedirect.com/science/article/pii/S0025712505705964

9. Klein I, Danzi S. Thyroid disease and the heart. Vol. 116, Circulation. 2007. p. 1725–35.

10. Mason JW. Myocarditis and dilated cardiomyopathy: An inflammatory link. Vol. 60, Cardiovascular Research. 2003. p. 5–10.

11. Canter CE, Simpson KP. Diagnosis and treatment of myocarditis in children in the current era. Circulation. 2014;129(1):115–28.

12. Midei MG, Dement SH, Feldman AM, Hutchins GM, Baughman KL. Peripartum Myocarditis and Cardiomyopathy [Internet]. Available from: http://ahajournals.org

## Supplementary Tables

| TUI | Semantic type description |
| --- | --- |
|  |  |
| T020 | Acquired Abnormality |
| T190 | Anatomical Abnormality |
| T049 | Cell or Molecular Dysfunction |
| T019 | Congenital Abnormality |
| T047 | Disease or Syndrome |
| T050 | Experimental Model of Disease |
| T033 | Finding |
| T037 | Injury or Poisoning |
| T048 | Mental or Behavioral Dysfunction |
| T191 | Neoplastic Process |
| T046 | Pathologic Function |
| T184 | Sign or Symptom |

Supplementary Table S1: List of UMLS type unique identifiers (TUIs) used to denote complaints.

*See file Supplementary table S2.xlsx*

Supplementary Table S2: Top 50 most significant complaints for each phenotype, ranked by p-value (smallest to largest). Significance was determined using a one-sided (greater) t-test with Bonferroni correction testing the null hypothesis that the distribution of values of TF-IDF features for a medical entity in cluster *i* are drawn from the same distribution as the same entity in all other clusters. Also shown are the percentage of patients in the cluster with the concept mentioned, as well as the percentage of patients in the overall cohort with mentions of the concept. If a cluster has less than 50 significant complaints, all are shown.

## Supplementary Figures

**

**

Supplementary Figure S1: Quantifying cluster stability. (A) Violin plot depicting the distributions of Jaccard index (left violin, cyan) and semantic similarity values (right violin, khaki) between original K-means clustering and clustering on a bootstrapped dataset subsampling 50% of the dataset (number of iterations = 1000). Diamonds mark the median of the distribution, while dotted lines denote the 25^th^/75^th^ quartiles. Local maxima for the Jaccard index/semantic similarity are marked with cyan/khaki stars. Green corridors show values of K for which the Jaccard index and semantic similarity are both local maxima, K ≤15. (B) Median values of the Jaccard index (top) and semantic similarity (bottom) between original K-means clustering and clustering on a bootstrapped dataset subsampling for fractions of data in [0.5, 0.25, …, 0.0078125].

*
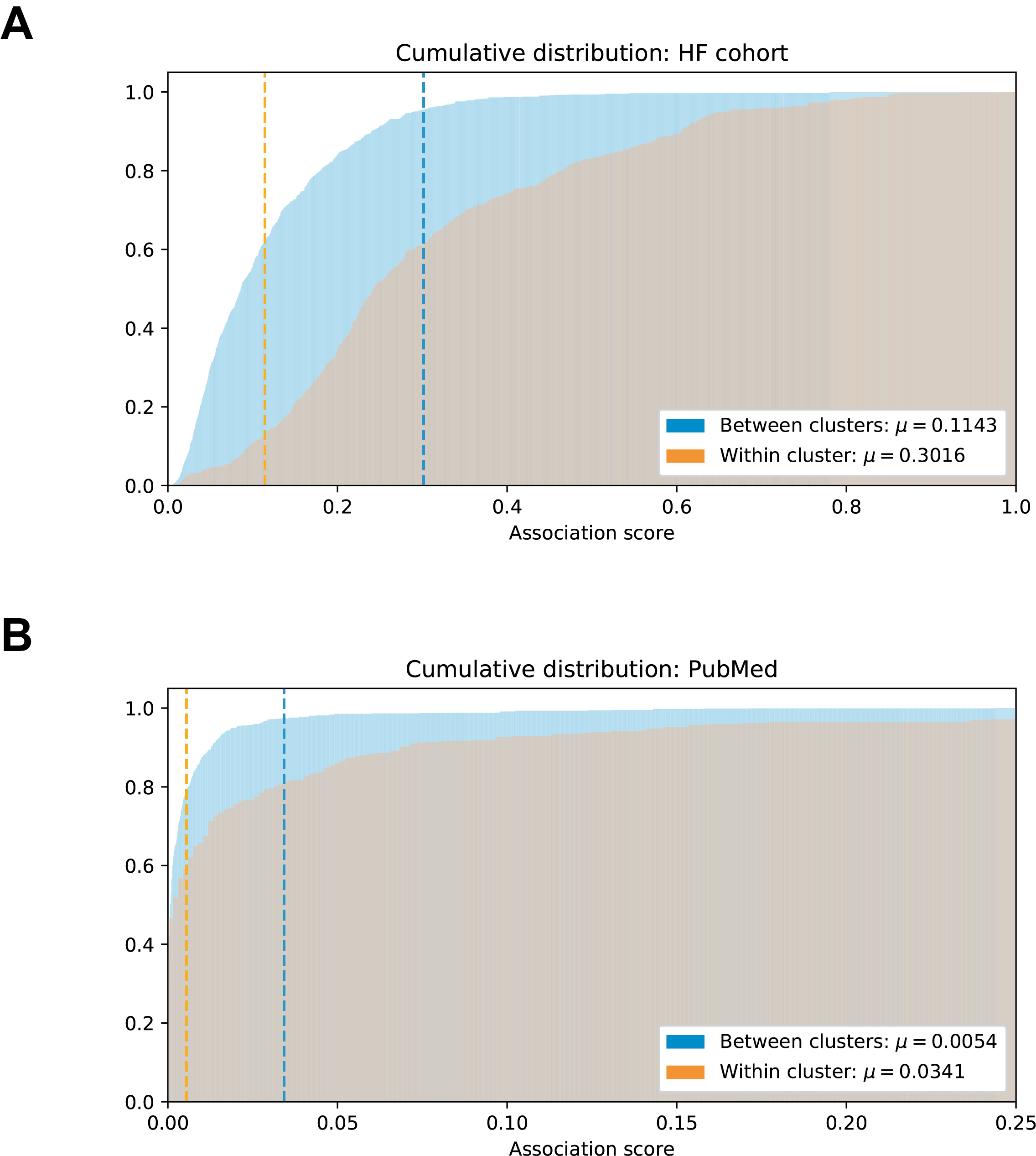
*

Supplementary Figure S2: Quantifying associations between HF complaints. (A) Cumulative distribution of association scores for concept pairs in the top 10 most significant (smallest p-value) concepts within each cluster (orange) and between each cluster (blue) within the HF cohort. Dotted lines show mean value. (B) Same as (A) for PubMed queries.


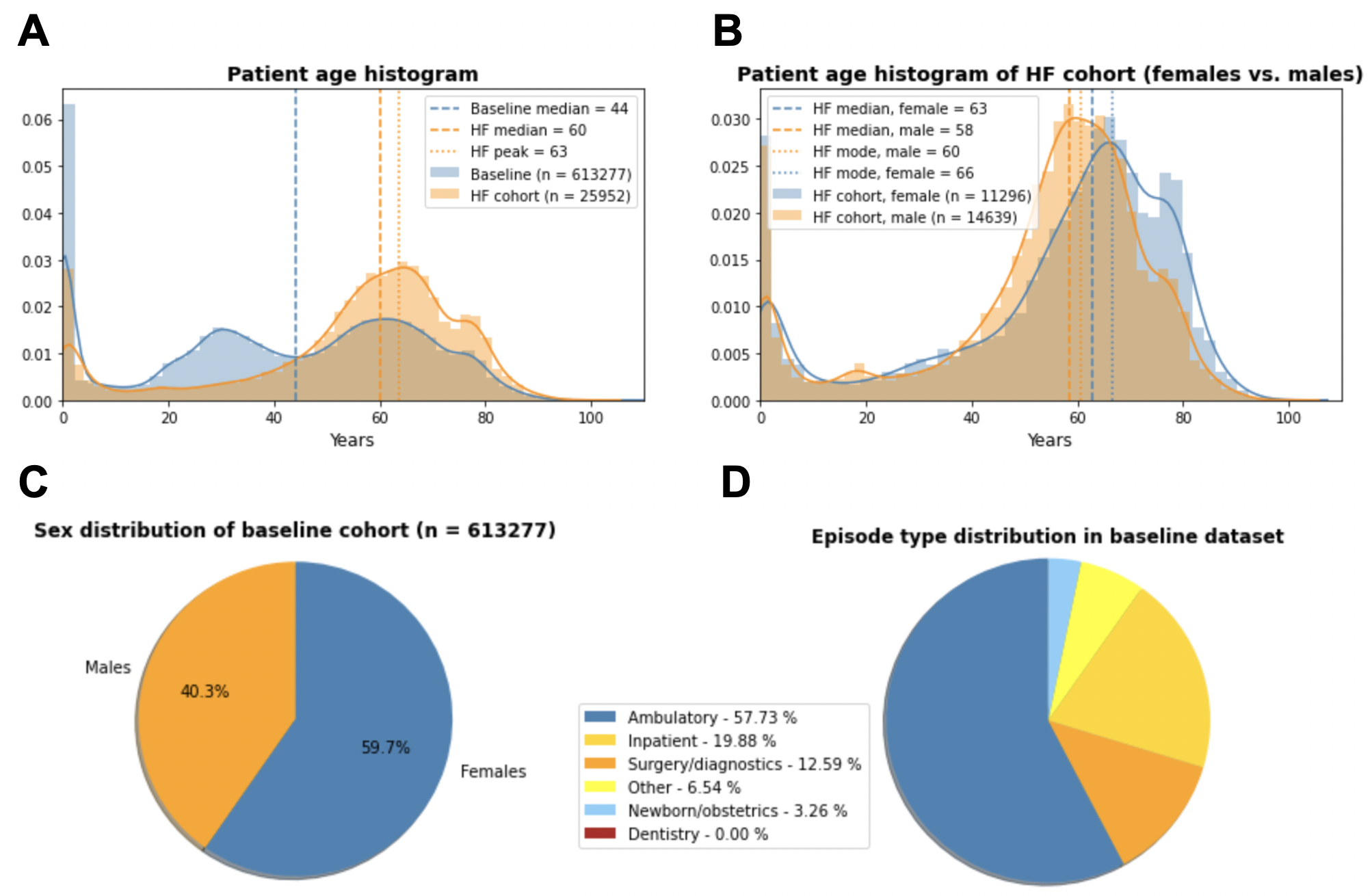


Supplementary Figure S3: (A) Normalized age distribution of all patients within the metropolitan center (n = 613,277) and the heart failure cohort (n = 25,952). The median age for the patient population within the entire metropolitan center is 44, while the heart failure cohort is older, with a median age of 60. The mode of the heart failure age distribution of patients is 63. (B) Comparison of age distributions of females vs. males in the heart failure cohort. The median and mode of each distribution are also shown. We observe that males within the heart failure cohort (median age = 58) are younger than females (median age = 63). (C) Sex distribution of the distribution of all patients within the metropolitan center (n = 613,277). (D) Distribution of episode types in the baseline dataset.
